# Cerebrospinal fluid opening pressure in clinical practice – a prospective study

**DOI:** 10.1007/s00415-020-10075-3

**Published:** 2020-07-17

**Authors:** Siri Hylleraas Bø, Christofer Lundqvist

**Affiliations:** 1 Department Neurology, Akershus University Hospital, Lørenskog, Norway; 2 Health Services Research Unit, Akershus University Hospital, Lørenskog, Norway; 3 University of Oslo, Inst. Clinical Medicine, Campus Akershus University Hospital, Lørenskog, Norway

**Keywords:** Idiopathic intracranial hypertension, Headache, Body mass index, Normal limits

## Abstract

**Background:**

Measurement of CSF opening pressure (CSFOP) is valuable and much used in the investigation of several neurological conditions. However, there are different opinions regarding reference values and influence of age, gender and body mass index (BMI). We have, in a previous study, noted possible differences in CSFOP between gender and age groups. Here the aim was to collect information regarding normal distribution of CSFOP in an out-patient sample and also include BMI.

**Methods:**

We collected CSFOP from a lumbar puncture, following a standardized procedure, performed in an ordinary neurological out-patient sample. Age, gender and BMI was also registered. Descriptive statistics and linear regression was used.

**Results:**

339 patients with a normal distribution of age and BMI were included consecutively (60% females). We found a mean CSFOP of 17.5 H_2_O (range 4.0–30.0). In multivariable linear regression, age, gender and BMI all independently affected CSFOP. Male gender (β = 1.5, *p* = 0.002), lower age (β =  – 0.095, *p* < 0.001) and higher BMI (β = 0.42, *p* < 0.001) were all associated with higher CSFOP.

**Conclusion:**

Using two standard deviations, we provide suggestions for CSFOP limits with respect to gender, age and BMI. Our results suggest that CSFOP cut-offs for pathological intracranial hypertension should be raised with these factors taken into consideration. As a “rule-of-thumb” we suggest the following cut-offs: for males < 30 cm H_2_O (< 25 if over age 70), and for females < 25 cm H_2_O (27.5 if over 30 BMI). A diagnosis of intracranial hypertension should not be given without such considerations.

## Introduction

Lumbar puncture and subsequent analyses of the cerebrospinal fluid (CSF) are important parts of many medical work-ups, particularly concerning possible pathological conditions in the central nervous system (CNS). Life-threatening conditions like bacterial meningitis and subarachnoid hemorrhage can quickly be diagnosed by lumbar puncture and allow treatment to be started without delay. In the investigation of several neurological conditions, lumbar puncture is a highly valuable supplement, and for some conditions like idiopathic intracranial hypertension it is the key diagnostic test, if a measurement of the cerebrospinal opening pressure is carried out correctly.

In order to interpret the findings of the cerebrospinal fluid opening pressure (CSFOP), however, several clinical factors must be taken into account. Firstly a level of what can be considered a normal upper CSFOP value must be established. In the literature there exist different opinions as to the limit of what is a normal CSFOP, whether all values outside the normal range should be considered pathological and what factors may influence the normal limits.

The lack of full knowledge of the variation in CSFOP could lead to a false conclusion of pathologically raised intracranial pressure, and give rise to unnecessary tests and examinations as well as anxiety and stress for the patient. There is also a risk for misinterpretation of a potential pathological CSFOP if other factors, such as age, gender and BMI, which may influence CSFOP are not considered along with the clinical findings.

The International Headache Society (IHS) defines increased CSFOP as above 25 cm H_2_O for the non-obese and above 28 for obese children, in the International Classification of Headache Disorders 2018 [13]. The limits of what is considered a normal CSFOP have been revised over the last two decades, and it was previously considered that 20 cm H_2_O was the upper limit in the IHS Classification from 2004 [1]***.***

In a previous prospective study, we collected CSFOP data from 348 patients in different clinical work-ups, including acute headache admissions, patients undergoing lumbar myelography, and outpatients from our neurology clinic [3]. The results from this study gave indications of a real difference in CSFOP between women and men, and also a tendency for mean CSFOP to become lower with increasing age, although the results were not statistically significant. We also observed that the range of mean CSFOP was as wide as 7.5 to 30 cm H_2_O, even when all patients with secondary causes for acute headache were excluded. As these results were suggestive of the need for new knowledge concerning CSFOP, we wanted to do an extended assessment of the CSFOP in a population closely resembling the general population.

In this prospective study we wanted to assess the influence of the factors age, sex, BMI on CSFOP in a neurological out-patient population closely resembling the general adult population, and in circumstances possibly less stressful than during an emergency admittance.

Our pre-formed hypotheses concerning CSFOP were:

That CSFOP up to at least 30 cm H_2_O can be measured, without necessarily implicating intracranial disease, indicating that the normal range of CSFOP values may need to be adjusted and that the CSFOP value itself not should be interpreted without reasonable concordance with symptoms and clinical findings.

That women in general have a lower base CSFOP than men, perhaps explaining the observed preponderance of women experiencing post-lumbar puncture headache.

## Material and methods

Akershus University Hospital has a catchment area of 500,000 inhabitants. The Neurology Department is the sole neurology specialist center in this area, and has extensive out-patient consultations. As part of the clinical work-up, as well as for research purposes, many patients are referred to lumbar puncture (LP) for analyses of the cerebrospinal fluid (CSF), and at our hospital a lumbar puncture in most cases includes a measurement of the CSF opening pressure (CSFOP).

We collected data from lumbar punctures performed at our out-patient clinic in the period 12.11.2013– 15.06.2017. These adult patients (18 years and above) were undergoing work-up for possible neurological conditions, and LP was part of the work-up. They were included consecutively and without regard to age, gender or clinical symptoms. As this was a planned procedure with an interval from weeks to months after consultation, the patients were rarely in acute pain or acute distress and anxiety.

All patients had been examined with CT scans or MRI of the head and brain previous to the LP, thus excluding possible expansive lesions or radiological signs of raised intracranial pressure.

Junior consultants working in the neurology department performed the LPs, following the standard procedure of the department; the patient positioned relaxed on his or her left side, legs slightly flexed, use of spinal needle 20G or 22G, and opening pressure measured at the beginning of the procedure, using a single-use manometer held at the same level as the needle and given time to reach steady-state.

The data collected were sex, age at time of LP, weight, height and CSFOP. The patients were divided into four age groups; younger than 30 years, between 31 and 50 years, between 51 and 70 years, and above 70 years. From the weight and height data, body mass index (BMI) was calculated. The patients were further divided into four BMI groups; BMI less than 20, BMI between 20 and 25, BMI between 25 and 30, and BMI over 30.

For statistical analyses we used standard descriptive tests (*χ*^2^ test for categorical and Student’s *t*-test and ANOVA for numerical data). Means and standard deviations or 95% confidence intervals (C.I.) are given. Linear regression was performed for bivariate and multivariate analyses of factors associated with CSFOP, for the multivariate analyses the covariates were entered stepwise. For all analyses, significance limits were set to *p* < 0.05.

All patients underwent LP as a necessary part of a diagnostic work-up, and the measuring of CSFOP did not represent any further discomfort or risk of complications. The patients gave informed consent, and the Regional Ethics Committee approved the study.

## Results

### Sample

We included 339 patients in our study, of these 339 patients 205 (60%) were women, and 134 (40%) were men.

The mean age was 46.2 years, and the age distribution was similar for the group of women and the group of men (Table 1). Thirty-three patients (9.7%) were younger than thirty years, 187 patients (55.2%) were between 30 and 50 years old, 97 patients (28.6%) were between 50 and 70 years old, and 22 patients (6.5%) were older than seventy years of age.

**Table 1:** Basic characteristics of sample

	Males *n* = 205, (60.5%)	Females *n* = 134 (39.5%)	Total	*p*-value (M vs. F)
Age (mean, S.D.)	50.7 (15.0)	43.3 (12.4)	46.2 (13.9)	< 0.001
BMI (mean, S.D.)	26.2 (3.54)	24.3 (3.98)	25.1 (3.9)	< 0.001

There was no significant difference between the sexes concerning distribution of BMI (Table 1). 34 patients (10%) had BMI less than 20, 169 patients (50%) had BMI between 20 and 25, 103 patients (30%) had BMI between 25 and 30, and 33 patients (10%) had BMI above 30.

### Cerebrospinal fluid opening pressure

**Fig. 1:**
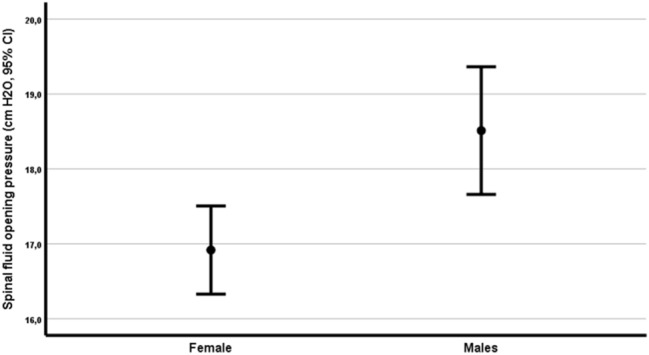
Mean spinal fluid opening pressure between genders

The mean CSFOP was 17.5 cm H_2_O, with the minimum value at 4.0 and the maximum value at 30.0, The mean CSFOP for women was 16.9, mean CSFOP for men was 18.5 (*p* = 0.003). (Fig. 1).

In univariate analyses, the CSFOP for men was significantly higher than the CSFOP for women (β = 1.6, *r*^2^ = 0.028, *p* = 0.002; Fig. 1 and Table 2).

**Table 2:** Univariate analyses of CSFOP for different age groups and BMI groups by gender and overall

	CSFOP males (S.D.)	CSFOP females (S.D.)	CSFOP total (S.D.)	*p*-values (M vs. F)
*Age groups*				
< 30	19.5 (4.8)	18.2 (3.8)	18.5 (4.0)	0.47
31–50	19.6 (4.6)	17.2 (4.2)	17.9 (4.5)	0.002**
51–70	18.3 (4.9)	15.8 (4.0)	17.2 (4.7)	0.007**
> 70	14.6 (5.1)	13.2 (5.5)	14.1 (5.1)	0.59
*BMI groups*				
< 20	19.4 (5.9)	15.0 (4.9)	15.6 (5.2)	0.172
20–25	17.5 (5.4)	15.8 (3.7)	16.4 (4.5)	0.034*
25–30	19.4 (4.4)	18.8 (3.4)	19.1 (3.9)	0.424
> 30	19.5 (4.6)	22.1 (2.7)	20.8 (4.0)	0.052

**Fig. 2:**
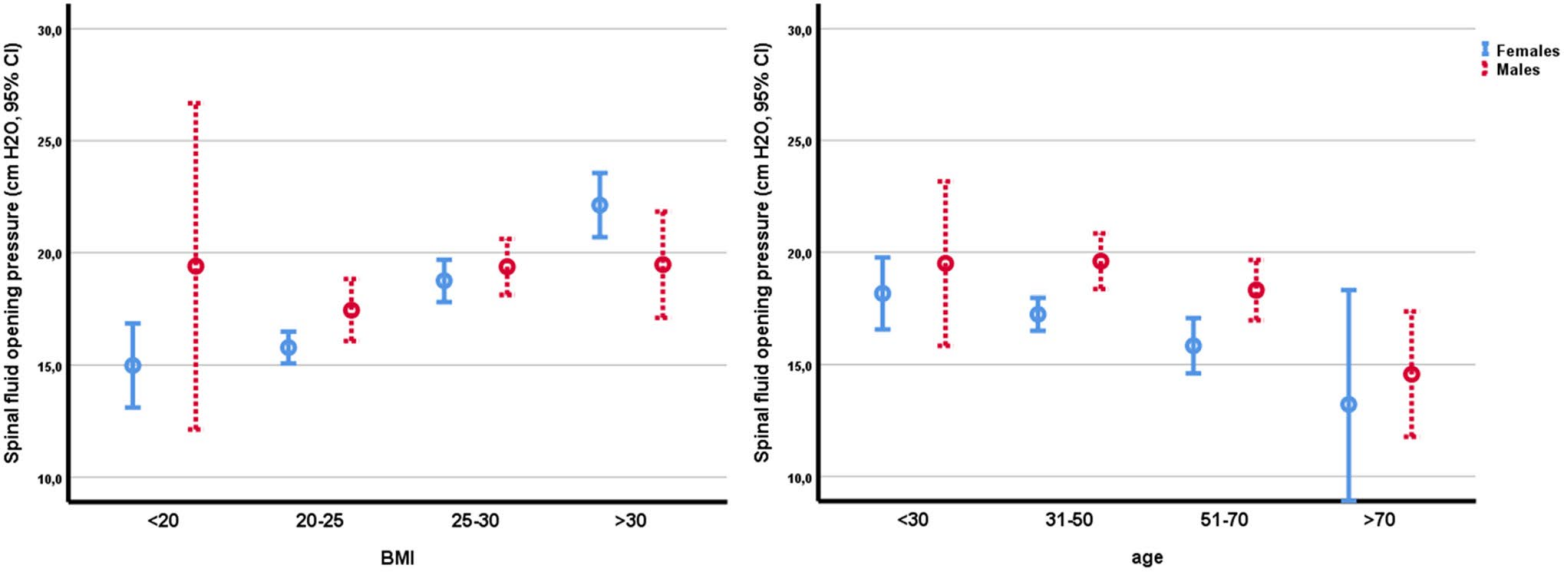
CSF opening pressure by Body mass index category **a** and by age **b** in females versus males

In univariate analyses, the CSFOP was reduced with increasing age (β =  – 0.065, *r*^2^ = 0.038, *p* < 0.001) and CSFOP increased with increasing BMI (β = 0.42, *r*^2^ = 0.13, *p* < 0.001; Fig. 2 and Table 2).

In a multivariate model with gender, age and BMI as covariates, all three variables were shown to be highly significant as predictors of CSFOP. Stepwise entered variables gave the following parameters: BMI β = 0.42, *p* < 0.001, age β =  – 0.095, *p* < 0.001, gender β = 1.5, *p* = 0.002; *r*^2^ = 0.21. Thus BMI was the strongest predictor but model fit improved also when stepwise entering both age and gender.

## Discussion

In the present study we made four significant findings. We were able to demonstrate that CSFOP levels above 20 cm H_2_O are frequent and do not always indicate a pathological condition. The same observation applies for levels above 25 cm H_2_O, and probably levels up to 30 cm H_2_O cannot invariably be considered pathological. This indicates that the range of CSFOP must be broadened in clinical practice. We found that CSFOP was significantly lower in women compared to men, became significantly lower as the age in the patient groups increased and higher as BMI increased. In multivariate analyses all three variables independently predicted CSFOP.

Regarding study limitations, we are aware that our study does not represent a true primary population sample. However, we suggest that it represents a typical neurological out-patient sample in Scandinavia and will therefore represent a useful comparison for patients in this setting. As previously suggested, patients recruited via the emergency room and undergoing spinal puncture based on suspected diagnoses such as thunderclap headache, may have stress- and pain-related elevation of CFOP and may therefore not be representative for the out-patient setting [3]. Therefore the present sample is suggested to be more adequate. Spinal punctures were made for a number of different reasons and none of our patients had a diagnosis of idiopathic intracranial hypertension, normal pressure or obstructive hydrocephalus and intracranial mass lesions were, in all cases excluded by a previous brain CT or MR. CSFOP pressure measurements were added routinely for the purpose of the present study. Puncture technique was standardised as described. Regarding measuring technique, Lehnfeldt et al. compared intracranial pressure (ICP) measured directly in brain tissue to assessments by LP in the same patients, and found LP with CSFOP measurement to be an accurate technique to determine ICP in supine patients with communicating cerebrospinal fluid system [11]. In order not to reduce consent and for ethical reasons, we decided not to collect any additional disease-related data and thus only collected data about age, gender and BMI. Though this may perhaps be criticized regarding questions of CSFOP in specific disease conditions, our aim was simply to collect overall data on CSFOP in our whole clinical population to serve as a basic guideline.

The relationship between age and CSFOP must be regarded as reasonably well established with several studies showing results similar to ours of reducing CSFOP with age [7]**.** May et al. [12] have also verified this in an experimental study measuring the production of CSF in 7 young and 7 elderly volunteers, and found that the CSF production was significantly lower in the elderly. A gender difference with lower CSFOP in women has also been clearly established [7]**.**

The question of BMI and its influence on CSFOP has been the subject of discussion and controversy. Our mean CSFOP of 17.5 cm H_2_O (median also 17.5) was very similar to that of Whiteley et al. who studied CSFOP in 242 neurological outpatients, and found a median of 17 cm H_2_O with a 95% confidence interval for distribution from 10 to 25. They concluded that CSFOP up to 25 should not be deemed abnormal in all cases, and that values up to 28 can be normal in some patients [14]. Based on previous research, a 2015 Lancet review also defines the upper limit of normal CSFOP as 25 cm H_2_O, but acknowledges that healthy individuals can have CSFOP up to 30 cm H_2_O and, occasionally even higher [5]. Some previous studies have also found BMI to be only weakly correlated to CSFOP and of little consequence in clinical practice [4, 14]. Bono et al. found no association between BMI and CSFOP when MR was normal. However, they also found no patients with a CSFOP over 20 cm H_2_O [4]. In the 2015 review, it is also stated that the effect of BMI on CSFOP is not significant [5].

In contrast, in the present study, where we also find CSFOP well above 20 cm H_2_O, BMI was the strongest factor predicting the measured CSFOP, with significantly increasing values with increasing BMI. Interestingly, looking at each gender separately, BMI category was a significant factor only for CSFOP of women. The mean BMI of our patients was 26.2 for men and 24.3 for women with a similar population distribution as in relevant normal populations [10]. Our results are partly supported by another large retrospective study of CSFOP compared to BMI and intraocular pressure. These authors found a direct, linear relationship between CSFOP and BMI, whereas there was no relationship between BMI and intraocular pressure. However, in contrast to us, they found no difference in CSFOP between men and women [2].

Our findings further support those of Fleischman et al. [7] who, in a large retrospective study of CSFOP in relation to age, sex, BMI, race and intraocular pressure, found that age, sex and BMI were all associated with differences in CSFOP. This study, though to our knowledge the largest performed so far, was retrospective and performed over a time period of over 10 years, therefore the standardization of the procedure is somewhat difficult to ascertain. Our prospective study with standardized procedures in our out-patient sample gave similar though somewhat higher (1–2 cm H_2_O) CSFOP values with a remarkably similar relationship with both age, gender and BMI. Interestingly, in the Fleischman study, intraocular pressure seemed to rise with decreasing CSFOP [7]. This relationship deserves further studies, not least considering publications concerning difficulty in diagnosing benign intra-cranial hypertension based on fundoscopy [6]***. ***In light of the high prevalence of primary headaches and the growing prevalence of obesity, it is important to avoid misdiagnosing of idiopathic intracranial hypertension with subsequent unnecessary examinations and treatments [6]. Recent diagnostic criteria for this headache entity suggest using 25 cm H_2_O as a cut-off for pathologically raised CSFOP [6, 9, 13]. Our studies as well as those of Fleischman [7] suggest that more differentiated cut-offs, taking into consideration both age, gender and BMI should be used. Fleischman and coworkers have, more recently, based on retrospective clinical data from the Mayo clinic as well as data from Xie and coworkers from Beijing, compared estimation of CSFOP using regression model derived formulae with measured CSFOP values [8, 15]. Though these models in addition to age and BMI also included blood pressure, model performance was similar to our results. In addition, using the Mayo derived formula on the Beijing population and vice versa gave poor prediction results with ICC values between 0.06 and 0.14. The authors warn against using generalized formula-derived CSFOP estimates especially between different populations. Our data thus provide prospectively measured CSFOP values for a Scandinavian population. Based on cut-offs of mean ± 2 standard deviations the suggested cut-offs based on our population are given in Table 3. Based on these tabulated values and as a “rule-of-thumb” we suggest upper cut-offs, i.e. limits for diagnosis of idiopathic intracranial hypertension in our population should be: for males < 30 cm H2O (< 25 if over age 70), and for females < 25 cm H2O (27.5 if over 30 BMI).

**Table 3:** Calculated cut-offs for normal CSF opening pressure for different age and BMI groups in our neurological out-patient population

	CSFOP Males	CSFOP Females	CSFOP Total
*Age groups*			
< 30	9.9–29.1	10.6–25.8	10.5–26.5
31–50	10.4–28.8	8.8–25.6	8.9–25.9
51–70	8.5–28.1	7.8–23.8	7.8–26.6
> 70	4.4–24.8	2.2–24.2	3.9–24.3
*BMI groups*			
< 20	7.6–31.2	5.2–24.8	5.2–26.0
20–25	6,7–28.3	8.4–23.2	7.4–25.4
25–30	10.6–28.2	12.0–25.6	11.3–26.9
> 30	19.3–28.7	16.7–27.5	12.8–28.8

Limits set by means ± 2 standard deviations

## Conclusion

The CSFOP is independently influenced by the patient`s age, gender and BMI.

The range of what is considered a normal CSFOP needs to be broadened, and must not be assessed independently from clinical findings, age, gender and BMI. We thus suggest raised CSFOP limits with attention to these variables. A diagnosis of intracranial hypertension should not be given without such considerations.

## Data Availability

Data is stored according to the Ethics board approval but anonymized data is available upon reasonable request to the authors.
